# Phase-Contrast Hounsfield Units of Fixated and Non-Fixated Soft-Tissue Samples

**DOI:** 10.1371/journal.pone.0137016

**Published:** 2015-08-31

**Authors:** Marian Willner, Gabriel Fior, Mathias Marschner, Lorenz Birnbacher, Jonathan Schock, Christian Braun, Alexander A. Fingerle, Peter B. Noël, Ernst J. Rummeny, Franz Pfeiffer, Julia Herzen

**Affiliations:** 1 Department of Physics & Institute of Medical Engineering, Technische Universität München, Garching, Germany; 2 Institute of Forensic Medicine, Ludwig-Maximilians-Universität, Munich, Germany; 3 Department of Diagnostic and Interventional Radiology, Technische Universität München, Munich, Germany; Argonne National Laboratory, UNITED STATES

## Abstract

X-ray phase-contrast imaging is a novel technology that achieves high soft-tissue contrast. Although its clinical impact is still under investigation, the technique may potentially improve clinical diagnostics. In conventional attenuation-based X-ray computed tomography, radiological diagnostics are quantified by Hounsfield units. Corresponding Hounsfield units for phase-contrast imaging have been recently introduced, enabling a setup-independent comparison and standardized interpretation of imaging results. Thus far, the experimental values of few tissue types have been reported; these values have been determined from fixated tissue samples. This study presents phase-contrast Hounsfield units for various types of non-fixated human soft tissues. A large variety of tissue specimens ranging from adipose, muscle and connective tissues to liver, kidney and pancreas tissues were imaged by a grating interferometer with a rotating-anode X-ray tube and a photon-counting detector. Furthermore, we investigated the effects of formalin fixation on the quantitative phase-contrast imaging results.

## Introduction

X-ray phase-contrast computed tomography (CT) is an emerging three-dimensional (3D) imaging modality based on a fundamentally different image formation process from that of conventional attenuation-based CT [1]. It may potentially benefit future clinical diagnosis by providing additional information and enhancing soft-tissue contrast [2, 3]. A promising approach to X-ray phase-contrast imaging is grating interferometry [4, 5]. Being operable at standard laboratory X-ray sources, grating interferometry may broaden the application of X-ray phase-contrast CT [6], enabling quantitative assessment of material properties within investigated samples [7, 8].

Because X-ray phase-contrast CT is not yet clinically available, research on biomedical phase-contrast imaging is currently conducted on excised and fixated tissue specimens [9–13]. The fixation step stabilizes the fine structural details of cells and tissues prior to examination, and is most commonly performed in aldehyde [14]. Although formalin fixation preserves tissues from decay, it induces intra- and inter-molecular cross-linking of macromolecules, thereby altering the physical characteristics of the tissues [15]. Consequently, the preliminary findings may considerably deviate from the in vivo scenarios, leading to incorrect impressions of the possible clinical benefits.

Hounsfield units (HU) are setup-independent numerical quantities that assist the differentiation of certain tissue types from medical X-ray CT data. A similar Hounsfield scale (HUp) has been defined for phase-contrast imaging [16]. The HUp directly assay the tissue electron density, a necessary parameter for realistic simulations and phantom design. Thus, HUp quantitation provides valuable insight into whether the phase-contrast method can adequately discriminate between tissues and satisfy the sensitivity demands of a phase-contrast CT system.

In this study, we quantified HUp in fixated and non-fixated tissues. The effects of formalin fixation on phase-contrast imaging were evaluated on porcine muscle, fat and rind samples. The samples were placed in physiological saline or varying concentrations of formaldehyde solutions and cooled to 4°C during the phase-contrast CT scan. The electron densities were then determined in non-fixated human tissues and compared with commonly used reference values tabulated in the literature. These investigations were conducted on muscle and adipose tissues, skin, tendon, brain and several internal organs (heart, liver, spleen, kidney and pancreas).

## Methods & Materials

### Grating-based phase-contrast imaging

As X-rays traverse an object, they undergo a phase shift. Among the several methods for measuring this phase shift, we selected X-ray grating interferometry for its compatibility with conventional X-ray sources and quantitative imaging capability. The typical Talbot–Lau interferometer installed at laboratory X-ray tubes consists of three gratings with micron-order periodicity. A phase grating creates periodic intensity modulations at certain distances in the beam direction. A sample placed in the beam path induces local shifts of this periodic intensity pattern. Since the pixel size of a standard detector usually exceeds the period of the pattern, an analyzer grating of the same period constructed from a highly absorbing material (in our case, gold) is mounted in front of the detector. This analyzer grating is translated perpendicularly to the beam over one grating period while several images are acquired. During this stepping process, the intensity of each detector pixel oscillates sinusoidally. The position of this curve can be evaluated by Fourier analysis and is directly associated with the refraction, i.e. the phase-shift, caused by the examined sample. Another highly absorbing gold grating is installed behind the X-ray tube and generates an array of many small, individually coherent sources. This grating ensures sufficient coherence, an important beam property for proper functioning of the interferometer. The principle of X-ray grating interferometry is detailed in Weitkamp et al. [4] and Pfeiffer et al. [6].

### Quantitative phase-contrast CT

In CT, many projection images are recorded from different angular directions and a 3D volume of the object is reconstructed using the filtered back projection algorithm. The phase-contrast projections obtained by X-ray grating interferometry are differential and an imaginary Hilbert filter is applied as filter function during reconstruction [17]. Correcting for setup-dependent factors such as grating spacings and periods, we can recover the 3D distribution of the refractive index decrement *δ* throughout the sample [7]. In clinical routine using conventional attenuation-based X-ray CT imaging, HU are well-established and simplify the diagnostics. Similar HUp in phase-contrast imaging are defined by [16]:

$$HUp=(\delta_{tissue}-\delta_{water})/(\delta_{water}-\delta_{air})\times 1000,$$ (1)

where *δ*_*tissue*_, *δ*_*water*_ and *δ*_*air*_ are the refractive index decrements of the image voxel (tissue), water and air, respectively. These units are energy-independent and are easily converted into corresponding electron densities [18]. The HUp of pure water is defined as 0 HUp. Positive and negative HUp values indicate electron densities above and below that of water (3.34 × 10^29^ e/m^−3^), respectively. Most of the quantitative analysis in our investigations is based on the HUp values, with some reference to electron densities.

### Experimental setup

**Fig 1:**
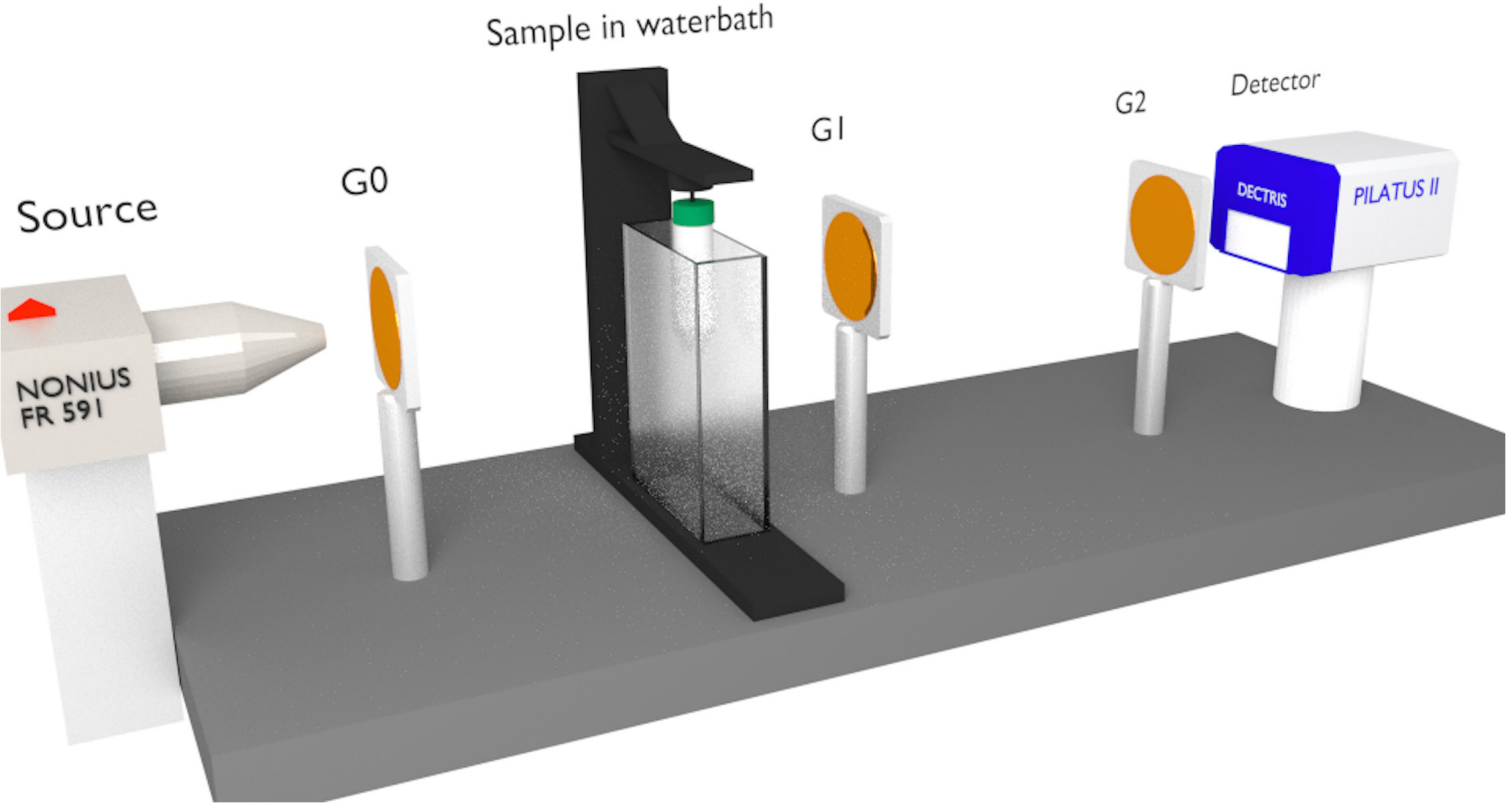
Illustration of the experimental phase-contrast computed tomography system. Three gratings with micrometer-sized structures are installed at a rotating anode X-ray tube (Nonius FR 591). The spatial resolution of the photon-counting X-ray detector (Pilatus II) is (172 × 172) μm^2^. Two gratings G1 and G2 together resolve the very small refraction angles induced by an object in the beam. Grating G0 (placed directly behind the source) guarantees proper functioning of the grating interferometer when operated under standard laboratory X-ray conditions. The investigated samples were mounted in front of G1 and submerged in a water bath during measurements to avoid image artefacts. The water was cooled to 4°C to decelerate the decay process of the soft tissues.

The measurements were conducted on a phase-contrast imaging system installed at the Physics Department of the Technische Universität München, Germany. This system combines a Talbot–Lau interferometer, a rotating molybdenum anode X-ray tube and a photon-counting imaging detector (Pilatus II, Dectris, Switzerland, 487 × 195 pixels, (172 × 172) μm² pixel area). The three gratings (each with a period of 5.4 μm) were fabricated at the Karlsruhe Institute of Technology (KIT) and by Microworks GmbH, Karlsruhe, Germany. The gold structures of the two highly absorbing gratings (one positioned behind the source, the other one in front of the detector) were approximately 60 μm high. The phase grating (with 9.5-μm-high nickel structures) was designed to introduce a *π* phase shift to X-rays of 27 keV, the effective energy of the polychromatic X-ray spectrum. This grating was placed equidistant (80 cm) to both gold gratings. By virtue of the long interferometer total length (160 cm), the system is highly sensitive to small differences in phase shift. Visibility, another important performance factor of the interferometer, is approximately 0.2. The rotation stage was mounted close to the phase grating, providing a sample magnification of 1.7. The field of view is of 4 × 2 cm^2^ and the effective pixel size is (100 × 100) μm^2^. A schematic of the imaging setup is presented in Fig 1.

### Sample preparation

**Fig 2:**
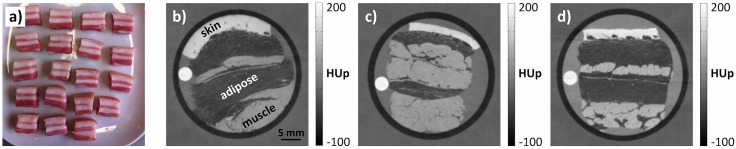
Representative phase-contrast imaging results of non-fixated and fixated tissue samples. (a) Photograph of porcine fat and rind samples on which the effects of formalin fixation were investigated (note the presence of skin, muscle and adipose tissues). Samples were immersed in physiological saline or fixated with formaldehyde solutions of varying concentrations. (b) Phase-contrast axial slice through a non-fixated porcine sample. Skin, muscle and adipose tissues are clearly differentiated by their signal intensities. (c) Phase-contrast image of a sample fixated in typical preservation solution (containing 3.7% formaldehyde). (d) Phase-contrast image of a sample fixated in 18.5% formaldehyde solution. The signal intensities in b) are visually indistinguishable from those of (c).

Three series of measurements were performed in this study. First, we investigated the differences in the quantitative HUp values of fixated and non-fixated tissue samples. We then examined the relationship between the obtained HUp values and formalin concentration. Finally, we determined the HUp values of non-fixated human tissue types. For the first two series, porcine fat and rind was obtained from the local butcher and cut into cubic pieces (approximately 2 cm each side). Representative samples are photographed in Fig 2(A). All samples include the rind (representing skin or connective tissue), two layers of fat (adipose tissue) and two layers of muscle tissue. The non-fixated samples were immersed in physiological phosphate buffered saline solution (PBS) and measured within 24 h of acquisition and preparation. The fixated samples were placed in containers filled with different concentrations of formaldehyde gas in water (1.85%, 3.7%, 7.4%, 12.3% and 18.5%) and incubated for approximately 2 weeks before measurement. The first series of measurements included five non-fixated samples of porcine fat and rind (in PBS) and five samples preserved in 3.7% formaldehyde solution (typical formaldehyde concentration of tissue fixative). The results of these measurements were used to identify the variances of a tissue’s HUp value among different samples and to quantify the magnitude of formalin induced changes. The second series of measurements was performed to get a better idea of how or why the formalin fixation may influence a tissue’s HUp value. Four additional porcine fat and rind samples were examined within this series, each of them prepared with a different concentration of formaldehyde gas in water (1,85%, 7.4%, 12.3% and 18.5%). The human tissue samples investigated within the third series of measurements were excised at the Institute of Forensic Medicine (Ludwig Maximilian Universität München, Germany) and approved by the Ethics Committee of the Faculty of Medicine of the Technische Universität München. The review board waived the need for consent. Two small pieces of the following tissue types were obtained: heart muscle, skin, tendon, liver, spleen, kidney, pancreas and brain. The heart muscle, skin and kidney samples further contained adipose tissue. The tissues were retained in PBS and stored in the refrigerator at 4°C. Each tissue type was scanned no later than three days after excision.

### Image acquisition and data analysis

For the measurements, each sample was placed in a plastic cylinder (diameter = 3 cm) surrounded either by PBS (non-fixated tissues) or formaldehyde solution at appropriate concentration (fixated tissues). Polymethylmethacrylate (PMMA) rods were inserted into the tubes to be able to cope with the polychromatic X-ray spectrum during the subsequent calibration of HUp. Throughout the measurements, samples were submerged in a water bath (thickness = 4 cm) to avoid beam hardening and phase wrapping artefacts that might affect the quantitative results [19, 20]. Furthermore, the water was cooled to 4°C to prevent decay of the non-fixated tissue samples.

A tomographic scan included 1200 projections consisting of 11 phase-step images, recorded with an exposure time of 3 s per image. The applied tube voltage was 40 kV and the tube current was set to 70 mA. The reconstructed 3D datasets give the refractive index decrements relative to the surrounding water (δ_tissue_ − δ_water_). These were converted into HUp by Eq (1), neglecting the air contribution. The PMMA rods enabled the assignment of effective energies to the measurements, by which we could look up the missing values of δ_water_ (remaining denominator in the equation). The calibration process is detailed in Willner et al. [18]. The final data were digitally stored in DICOM format and analyzed using OsiriX 4.0 (32 bit). To quantify the HUp values, ten regions-of-interest were selected within each tissue type for each measurement, and their averages were determined. The mean value and standard deviation of the ten averages were calculated.

## Results

### Comparison of fixated and non-fixated tissue values

Representative tomographic phase-contrast imaging results of a non-fixated and two fixated (in 3.7% and 18.5% formaldehyde solution) porcine samples are displayed in Fig 2B–2D. The different tissue layers are clearly distinguishable in all three axial slices. Skin yields the brightest signal, adipose tissue appears darker than the surrounding water and muscle tissue shows intermediate brightness. The PMMA rods appear as small white circular areas to the left of the porcine fat and rind. Visually, the non-fixated and fixated samples are very similar. The chief difference is that formalin stiffens the fixated samples, preventing their conformation to the plastic cylinder as observed for non-fixated tissues. The image noise in all measurements is approximately 3 HUp.

**Fig 3:**
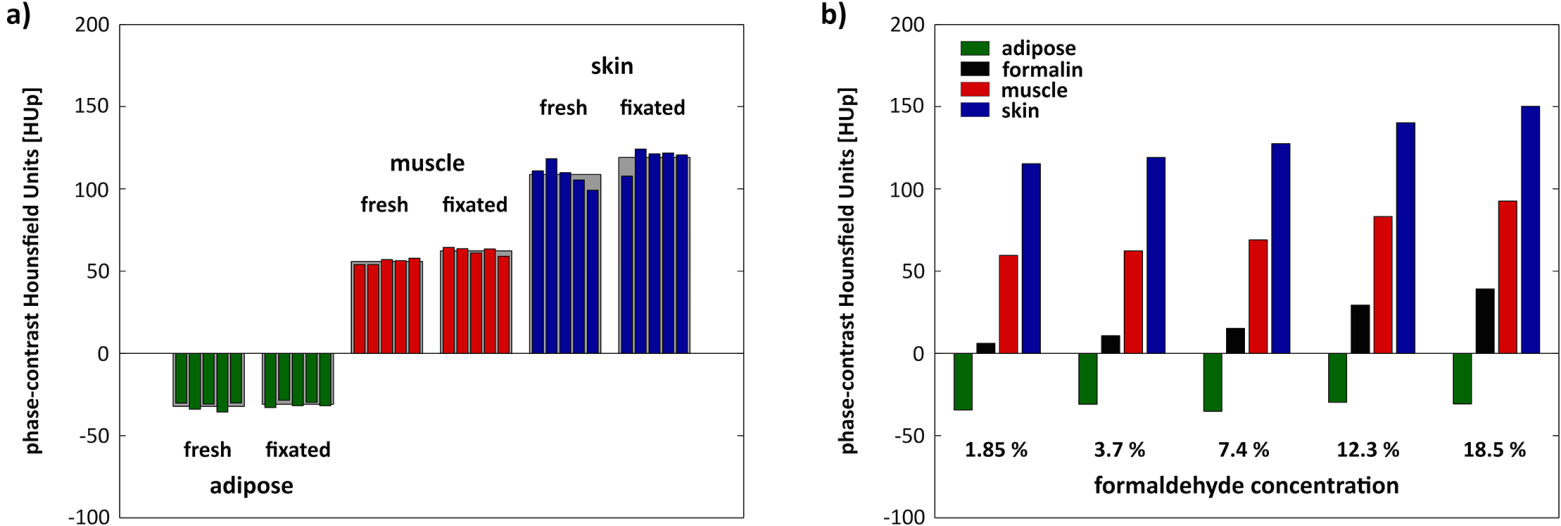
Graphical representation of the quantitative analysis of non-fixated and fixated tissue samples. (a) Comparison of phase-contrast Hounsfield units evaluated for five non-fixated porcine samples, and five samples fixed in 3.7% formaldehyde solution. Formalin fixation does not affect adipose tissue but increases the Hounsfield units of muscle and skin by approximately 10%, most likely by tissue shrinkage. (b) Phase-contrast Hounsfield units of the three investigated tissue types fixated at different formaldehyde concentrations. Again, adipose tissue is insensitive to formaldehyde concentration, but the HUp of muscle and skin follow the same trend as the actual formaldehyde solutions. This effect is due to replacement of water by the formaldehyde solution within the tissues.

The HUp values of the three tissue types obtained for the five non-fixated (PBS) and five fixated (3.7%) samples, and their corresponding means, are listed in Table 1. Fresh and fixated adipose tissue ranges from −30.1 to −35.7 HUp and from −28.5 to −33 HUp, respectively, with respective means of −32.2 HUp and −31.0 HUp. The difference is within the image noise, suggesting that fixation induces marginal changes in the quantitative tissue values. In contrast, fixation affects the values of muscle tissue and skin. In both cases, fixation increases the HUp values by approximately 10% (55.8 HUp versus 62.3 HUp in muscle tissue and 108.8 HUp versus 119.1 HUp in skin). The graphical overview in Fig 3(A) provides a quick visual assessment of these results.

**Table 1:** HUp values of five non-fixated (fresh) and five fixated (3.7% formaldehyde solution) tissue samples containing adipose tissue, muscle tissue and skin. The numbers in regular typeface represent the mean values and standard deviations (round brackets) obtained from ten regions-of-interest that were analysed for each of the three tissue types and each individual sample. The bold numbers finally give the mean values and standard deviations (square brackets) of these individual results and reflect the differences in the tissues’ HUp in non-fixated and fixated state.

	adipose	muscle	skin
fresh sample 1	−30.3 (3.3) HUp	53.9 (2.0) HUp	111.0 (4.0) HUp
fresh sample 2	−34.0 (4.0) HUp	54.0 (3.4) HUp	118.4 (5.3) HUp
fresh sample 3	−30.8 (3.0) HUp	57.0 (3.3) HUp	109.9 (4.4) HUp
fresh sample 4	−35.7 (1.2) HUp	56.4 (3.8) HUp	105.4 (5.1) HUp
fresh sample 5	−30.1 (2.9) HUp	57.9 (3.0) HUp	99.2 (3.6) HUp
fixated sample 1	−33.0 (3.0) HUp	64.4 (2.9) HUp	107.8 (5.8) HUp
fixated sample 2	−28.5 (3.0) HUp	63.7 (2.2) HUp	124.2 (3.2) HUp
fixated sample 3	−31.8 (3.8) HUp	61.0 (3.7) HUp	121.2 (3.2) HUp
fixated sample 4	−29.8 (1.7) HUp	63.5 (3.0) HUp	121.8 (4.5) HUp
fixated sample 5	−31.9 (0.9) HUp	59.1 (1.2) HUp	120.6 (2.4) HUp
**fresh samples**	**−32.2 [2.5] HUp**	**55.8 [1.8] HUp**	**108.8 [7.1] HUp**
**fixated samples**	**−31.0 [1.8] HUp**	**62.3 [2.2] HUp**	**119.1 [6.5] HUp**

Table 1 further displays the standard deviations that were calculated during the data handling. The standard deviations of the ten regions-of-interest that were analysed for each tissue type and measurement are stated in round brackets. They illustrate the variance of a tissue’s HUp within one sample. The standard deviations of the five mean values that were determined for a certain tissue type in the non-fixated and fixated case are given in square brackets. These numbers represent the variance of a tissue’s HUp among different samples. Most standard deviations are in the order of image noise (3 HUp) or smaller, implicating that uncertainties arising from tissue variability can be assumed to be low. Higher standard deviations are obtained for skin.

### Influence of formaldehyde concentration on quantitative HUp values

In addition to the five samples fixated with 3.7% formaldehyde solution, four porcine pieces fixated at different formaldehyde concentrations (1.85%, 3.7%, 7.4% and 18.5%) were examined to get a clearer picture of the reasons for the formalin induced changes. The determined HUp values of fat, muscle and rind at each formaldehyde concentration are presented in Table 2. For the case of no fixation and 3.7% formaldehyde concentration, the mean values of the fives samples from the first series are listed. The HUp values of the respective PBS or formaldehyde solutions are added to the analysis. The results are further illustrated in Fig 3(B).

**Table 2:** Effect of formaldehyde concentration on the quantitative phase-contrast Hounsfield unit of adipose tissue, muscle tissue and skin. For higher formaldehyde concentrations, the increase in the Hounsfield unit of muscle tissue and skin correlates with the Hounsfield unit of the formaldehyde solution itself.

	adipose	muscle	skin	PBS/formalin
**no fixation (PBS)**	−32.2 HUp	55.8 HUp	108.8 HUp	8.8 (HUp
**1.85% formaldehyde fixation**	−34.4 HUp	59.6 HUp	115.3 HUp	6.1 HUp
**3.7% formaldehyde fixation**	−31.0 HUp	62.3 HUp	119.1 HUp	10.8 HUp
**7.4% formaldehyde fixation**	−35.3 HUp	69.0 HUp	127.5 HUp	15.2 HUp
**12.3% formaldehyde fixation**	−29.8 HUp	83.2 HUp	140.2 HUp	29.3 HUp
**18.5% formaldehyde fixation**	−30.8 HUp	92.6 HUp	150.2 HUp	39.1 HUp

The quantitative HUp values of adipose tissue are insensitive to formaldehyde concentration whereas the HUp values of muscle tissue and skin are increasing with higher formaldehyde concentration. The measured HUp values of the formaldehyde solutions themselves follow the same trend; increasing from 6.1 HUp at 1.85% formaldehyde to 39.1 HUp at 18.5% formaldehyde. On further examination, the HUp values of muscle tissue and rind are directly correlated with the HUp values of the formaldehyde solution. If the values are adjusted for this effect, they are very similar at all five formaldehyde concentrations: 53.5, 51.5, 53.8, 53.9 and 53.5 HUp in muscle tissue and 109.2, 108.3, 112.3, 110.9 and 111.1 HUp in skin.

The porcine fat and rind sample fixated at the highest formaldehyde concentration (18.5%) was transferred to phosphate buffered solution after the first scan and re-measured several days later. In this measurement, the HUp values were altered to −30.2 HUp (up from −30.8 HUp) in adipose tissue, 61.8 HUp (down from 92.6 HUp) in muscle and 118.8 HUp (down from 150.2 HUp) in rind. Evidently, the elevated HUp values of muscle tissue and rind in formaldehyde solution were negated after incubation in PBS. However, the HUp values of both tissue types are approximately 10% higher than those of their fresh counterparts, and nearly identical to those of samples fixated with 3.7% formaldehyde.

### Phase-contrast HUp of non-fixated human tissues

**Fig 4:**
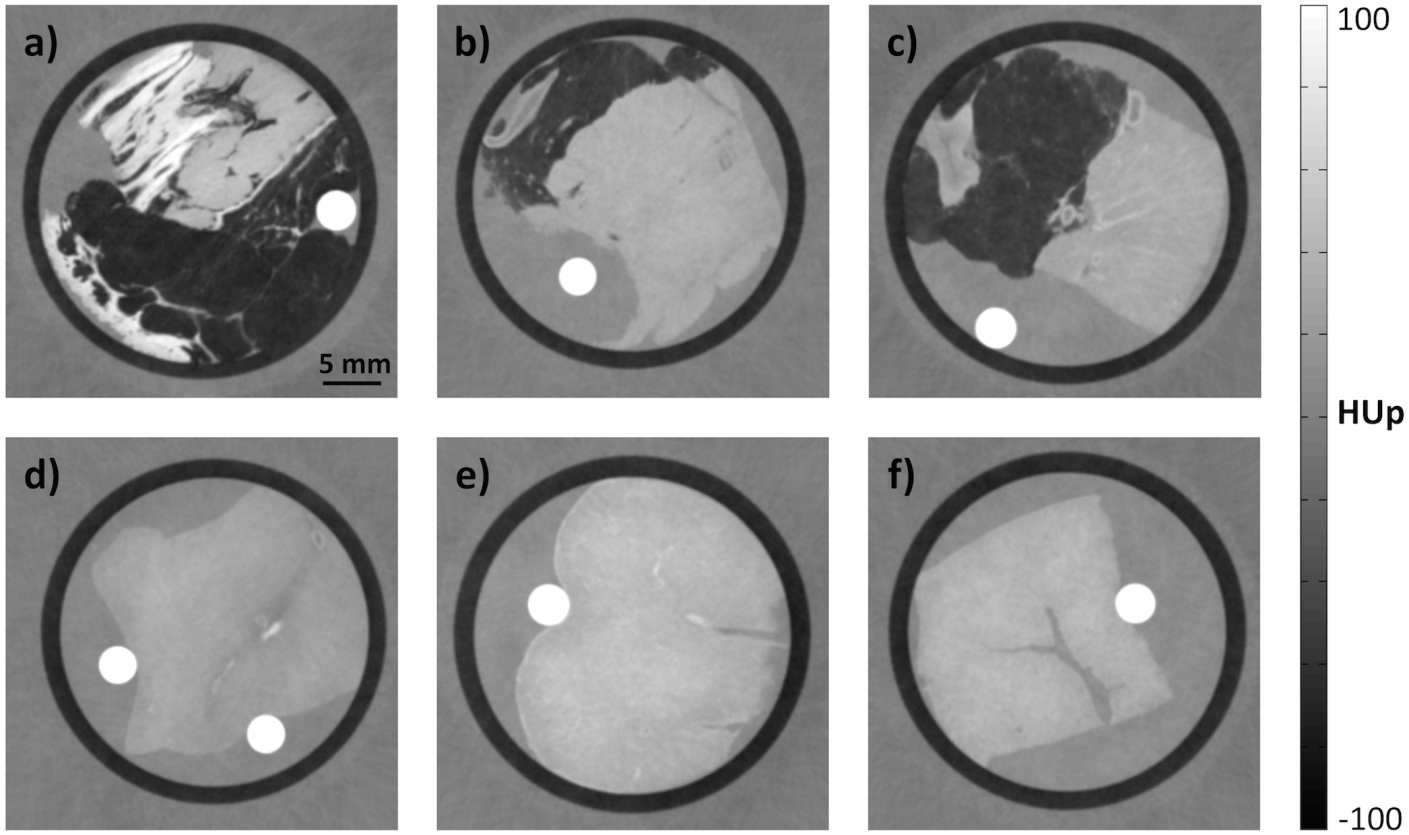
Representative phase-contrast imaging results of non-fixated human tissue samples. (a) Sample comprising tendon (bright, top), skin (bright, bottom), muscle (medium signal intensity) and adipose tissue (dark structures). (b) Heart muscle tissue with portions of fatty tissue. (c) Renal and adipose tissue. (d) Brain tissue. (e) Spleen tissue. (f) Piece of liver tissue. In each image, the high-intensity circular areas are the PMMA rods used to calibrate the quantitative phase-contrast Hounsfield units.

Fig 4 displays six representative tomographic phase-contrast images of human soft tissue samples. The evaluated HUp values of all investigated tissue types are presented in Table 3. Again, the standard deviations of the ten regions-of-interest’s averages are listed (round brackets) and give an idea of the variations within one tissue. For comparison, the literature values calculated from electron densities, previously published by Woodard and White [21], are also listed. Woodard and White recorded three different densities for some tissue types, reflecting the high variance in their results of which Table 3 lists the minimum and maximum. The data of Woodard and White are suggested as reference values by the International Commission on Radiation Units and Measurements [22], and underlie many theoretical considerations such as X-ray dose calculations or simulations of phase-contrast imaging.

**Table 3:** Phase-contrast Hounsfield units of various human soft tissue types evaluated from measurements and calculated from tabulated literature data. Ten regions-of-interest were analysed for each tissue type and measurement. The listed numbers are the mean values and standard deviations (round brackets) of the regions’ averages.

	sample 1	sample 2	literature (W&W)
**muscle**	37.0 (2.2) HUp	40.7 (3.2) HUp	39.4 / 40.4 HUp
**adipose**	−42.4 (3.9) HUp	−45.3 (3.2) HUp	−30.6 / −66.6 HUp
	−51.3 (1.8) HUp	−48.0 (5.0) HUp	
	−55.7 (4.1) HUp	−63.4 (4.6) HUp	
**skin**	93.8 (6.7) HUp	74.3 (9.5) HUp	78.0 HUp
**liver**	53,8 (1.8) HUp	41.7 (1.8) HUp	41.2 / 59.2 HUp
**spleen**	44.1 (3.3) HUp	45.9 (2.7) HUp	52.1 HUp
**kidney**	32.9 (1.7) HUp	32.1 (4.7) HUp	40.3 / 42.2 HUp
**pancreas**	26.4 (2.4) HUp	34.3 (3.1) HUp	34.1 HUp
**tendon**	66.5 (4.7) HUp	90.1 (7.2) HUp	101.6 HUp
**brain**	29.1 (1.7) HUp	32.9 (2.3) HUp	34 / 35 HUp

In the present investigation, the HUp values of muscle tissue are most consistent with the literature values. The two heart muscles yielded values of 37 HUp and 40.7 HUp. The corresponding literature values are approximately 40 HUp. The deviation from literature is less than 3 HUp (less than 1 e/nm^3^ in the electron density picture). Six HUp values were obtained for adipose tissue; two each from the heart, skin and kidney samples. The HUp values of adipose tissue range from −42.3 HUp to −63.4 HUp, within the range reported in the literature (−30.6 HUp to −66.6 HUp). The HUp results of liver are 53.8 HUp and 41.7 HUp, which are also comparable to the literature values (41.2 HUp to 59.2 HUp). The single electron density of skin reported by Woodward and White (78 HUp) is intermediate between our experimental results of 74.3 HUp and 93.8 HUp. The determined HUp values of the inner organs (spleen, kidney and pancreas) are up to 10 HUp lower than their corresponding literature values: 44.1 HUp and 45.9 HUp versus 52.1 HUp for spleen, 32.9 HUp and 32.1 HUp versus 40.3–42.2 HUp for kidney and 26.4 HUp and 34.3 HUp versus 34.1 HUp for pancreas. Furthermore, our experiments yielded lower HUp values for tendon and brain tissue: 66.5 HUp and 90.1 HUp versus 101.6 HUp for tendon; 29.1 HUp and 32.9 HUp versus 34/35 HUp for brain tissue.

## Discussion

Formaldehyde fixation is a complex process initialized by rapid penetration that stops autolysis, followed by covalent bonding and cross-linking with proteins [15, 23]. This process causes stiffening and shrinkage of the tissue sample. The reported shrinkage of excised skin specimens (size change from ex vivo to post-fixation) ranges from minimal to around 10% [24–27]. Substances such as carbohydrates, lipids and nucleic acids become trapped in the matrix of cross-linked protein molecules, but are not chemically changed by formaldehyde unless fixation is prolonged for several weeks [14, 28].

Comparing non-fixated porcine samples incubated in PBS with samples fixated in 3.7% formaldehyde, the HUp values of rind (skin) and muscle were elevated by 10% in the latter. The corresponding gain in electron density (~0.5%–1%) is probably caused by tissue shrinkage. This small density response compared to the large size reduction is attributed to the large amounts of water within tissues, which is displaced but not compressed during the shrinking process. The electron density increases mainly because the existent proteins become more closely packed within the tissues.

The quantitative HUp values of muscle and skin tissues are also affected by the replacement of water with formaldehyde solution in fixated samples. The electron density of 3.7% formaldehyde solution is similar to that of physiological PBS (337.6 versus 336.9 e/nm^3^), and thus exerts little impact on the HUp values. At higher formaldehyde concentrations, the solution electron densities rise from 339.1 e/nm^3^ (7.4% formaldehyde) to 343.8 e/nm^3^ (12.3%) to 347.1 e/nm^3^ (18.5%). The electron densities of muscle tissue and rind fixated with the same concentrations of formaldehyde solution increase by nearly the same amount.

Apparently, formalin fixation elevates the quantitative HUp values (i.e. electron densities) of soft tissues in two ways. First, the protein cross-linking shrinks the tissues and increases their protein density. Second, the water in the tissues is replaced by the penetrating formalin. This idea is supported by the results of the porcine sample initially fixated with 18.7% formaldehyde solution, and then incubated for several days in PBS. The HUp values of muscle and rind significantly decreased after PBS incubation to levels comparable with fixation in 3.7% formaldehyde, but remained 10% higher than those of unfixed tissue samples incubated in PBS. This indicates that the 10% increase in HUp is related to closer packing of the proteins.

Adipose tissue was insensitive to fixation at any formaldehyde concentration, probably because its high lipid content confers resistance to formalin within the first two weeks of fixation.

The HUp values of non-fixated human tissue types measured in this study are consistent with the literature values. This confirms that commonly used reference values are suitable for simulations, phantom design and dose calculations. Unfortunately, the literature values cannot capture the different compositions of diverse tissue types, and theoretical approaches are limited to universally classified tissue types. Consequently, simulation studies may not explore the full potential of phase-contrast imaging, and may therefore overlook interesting applications [18].

Some of the measured HUp values were considerably lower (by up to 10 HUp) than their literature equivalents. Expressed in electron densities, this deviation is small (approximately 1%), and is attributed either to the natural spectrum of tissue densities or to post-mortem tissue decomposition. Extending our results to an in vivo scenario, the measured HUp values may also be influenced by lack of blood flow in a tissue’s vascular system and the shrinkage induced by tissue excision [24].

Non-fixated tissues are very soft and buoyant what makes it difficult to prevent them from slightly moving in the surrounding liquid throughout the measurement. These positional inaccuracies cause blurring in some of the phase-contrast images. Although these motion artefacts do not influence the HUp values, they reduce the visibility of fine tissue structures such as collagen strands.

## Conclusions

Fixated tissue samples are easier to handle than non-fixated (fresh) tissue samples. Since the tissues are preserved, they can be subjected to longer storage and scanning times without cooling. Additionally, because the samples are hardened by the preservation, they remain fixed throughout the measurements, enabling sharp image acquisition. Although formalin fixation increases the electron densities of some tissue types, it does not degrade or artificially enhance the resulting image contrast and, thus, is suitable for most biomedical phase-contrast investigations.

In this study, the quantitative data obtained from various non-fixated human tissue types were largely consistent with electron densities tabulated in the literature. The literature values are accepted as reference values in phase-contrast simulations and in designing dedicated phantoms. To extend the database beyond standard tissue types and thereby explore the full potential of phase-contrast imaging, further quantitative investigation is required.
